# Thoroughbred

**Published:** 1882-01

**Authors:** 


ANSWER TO CORRESPONDENT.—J. N. N. is informed that the term thoroughbred refers to a distinct breed of race horses, who trace their descent from the Arabian running horse, and the term is only applied to such. We cannot say a thoroughbred Clydesdale or Perchon, but simply pure bred Clydesdale. The term is not applied to horned cattle.

